# In Silico Analysis of Possible microRNAs Involved in the Pathogenesis of White-Nose Syndrome in *Myotis lucifugus*

**DOI:** 10.3390/ijms26178200

**Published:** 2025-08-23

**Authors:** Anouska Agarwal, Craig K. R. Willis, Anuraag Shrivastav

**Affiliations:** 1 Department of Biology, The University of Winnipeg, Winnipeg, MB R3B 2E9, Canada; 2 Paul Albrechtsen Research Institute, CancerCare Manitoba, Winnipeg, MB R2H 2A6, Canada

**Keywords:** *Pseudogymnoascus destructans*, fat metabolism, hibernation, wildlife diseases

## Abstract

Since 2007, white-nose syndrome (WNS), caused by the fungus *Pseudogymnoascus destructans*, has killed millions of bats across North America by disrupting hibernation cycles, causing premature fat depletion and starvation. Little brown bats (*Myotis lucifugus*) from some populations persisting after WNS store larger pre-hibernation fat reserves than bats did before WNS, which may help bats survive winter starvation and mount an immune response to *Pd* in spring. MicroRNAs (miRNAs) are highly conserved, small, non-coding RNA molecules that regulate gene expression post-transcriptionally. Aberrant miRNA expression can affect metabolic pathways in mammals and has been linked to various diseases. If fat reserves and immune mechanisms influence survival from WNS, then miRNAs regulating metabolic and immune-related genes might affect WNS pathogenesis and bat survival. A previous study identified 43 miRNAs differentially expressed in bats with WNS. We analyzed these miRNAs for their roles in metabolism and immune-related pathways, using DIANA Tools and KEGG analysis, to determine a subset that could serve as biomarkers of pathophysiology or survival in WNS-affected bats. We identified miR-543, miR-27a, miR-92b, and miR-328 as particularly important because they regulate multiple pathways likely important for WNS (i.e., immune response, lipogenesis, insulin signaling, and FOXO signaling). As proof-of-concept, we used reverse transcription quantitative real-time PCR (RT-qPCR) to quantify the prevalence of these miRNAs in plasma samples of bats (*n* = 11) collected from a post-WNS population during fall fattening. All the selected miRNAs were detectable in at least some bats during fall fattening although prevalence varied among miRNAs. Future in vivo validation studies would help confirm functional roles and biomarker utility of these miRNAs for WNS-affected bats.

## 1. Introduction

Emerging infectious diseases of wildlife present a growing threat to biodiversity and human public health [1] and fungal pathogens have caused drastic declines in wildlife populations worldwide [2]. One such infectious disease, white-nose syndrome (WNS), is caused by the fungal pathogen, *Pseudogymnoascus destructans* (*Pd*) [3], and has led to devastating mortality in North American bats [4]. Three species of bats are now listed as endangered in Canada due to WNS [5], including the little brown bat (*Myotis lucifugus*), which, prior to the disease, was likely the most abundant and widespread bat species in North America [6]. WNS is named for the growth of visible white fungus on the muzzle, ears and wings of bats, and was first discovered in New York State in the winter of 2006/2007 [7]. *Pd* is a cold-tolerant fungus restricted to cold, humid and dark environments, and cannot grow above a maximum temperature of 20 °C or below 4 °C [8]. The fungus grows on the exposed skin of bats when they enter a hibernaculum for winter [7].

Hibernation is made up of bouts of torpor, a metabolically depressed state of reduced body temperature. Torpor allows hibernators to save enormous amounts of energy but, in bats, provides the ideal growth temperatures for *Pd*. During hibernation, mammals also downregulate immune function [9,10], which may further increase the susceptibility of some bat species to *Pd* [11,12]. Torpor bouts are interspersed with periodic arousals to normal body temperature, which appear to restore homeostasis and, for healthy bats, occur every few weeks [13]. Infection with *Pd* causes bats to arouse from torpor too frequently, likely due, in part, to dehydration caused by fluid loss from lesions in the highly vascularized flight membranes [14,15,16,17].

Torpor saves energy, but bats must still accumulate fat reserves before entering hibernation and, from mid-August to mid-September, their body mass increases dramatically [18,19]. For many populations, food is unavailable in winter with no way to replenish fat reserves during hibernation. Therefore, pre-hibernation fattening is crucial for survival, especially for bats facing added winter energy expenditure from WNS. Fat reserves appear to play a role in WNS survival, and evidence suggests that increased fat stores contribute to the survival of little brown bat populations affected by WNS [20,21,22,23]. Thus, understanding the mechanisms and pathways involved in energy metabolism and fat storage in bats is important for understanding the pathogenesis of WNS and bat survival. Pancreatic hormones, insulin and glucagon regulate blood glucose levels and, therefore, have a strong influence on energy storage and feeding. Insulin, a peptide hormone produced by beta-cells of the pancreatic islets, is released in response to rising blood glucose levels after feeding. It reduces plasma glucose by promoting its conversion into glycogen or fat, primarily through the phosphatidylinositol 3-kinases (PI3K)/protein kinase B (AKT) pathway. Activation of the PI3K/Akt pathway promotes lipogenesis by upregulating transcription of hepatic steroid regulatory element-binding protein (SREBP)-1c, essential for adipocyte differentiation and lipogenesis [24].

In fall, hibernators like little brown bats exhibit behaviors such as hyperphagia (increased appetite) along with hyperinsulinemia (high insulin levels) and insulin resistance, all of which contribute to fat accumulation [25]. Concurrently, leptin, a satiety hormone, initially increases with body mass and adiposity but eventually declines. This suggests a disconnect between leptin production and adiposity despite continued increases in fat accumulation [26]. As fall progresses, although pancreatic insulin levels rise, plasma insulin concentration drops, suggesting that insulin is used rapidly to enhance fat storage in preparation for hibernation. However, during hibernation, there is a notable reduction in insulin response. This diminished insulin activity suggests that the body shifts to using fat reserves accumulated during fall as a primary fuel source [27]. A significant reduction in phosphorylated Akt (p-Akt) during torpor further supports this shift [28]. Phosphorylation of Akt promotes lipid synthesis by activating SREBP1c [29]. While p-Akt typically supports lipid synthesis, its reduction during torpor reduces lipogenesis and allows for the mobilization and use of fall fat reserves for energy. In bats affected with WNS, this balance seems to be disrupted, causing increased arousal frequency during hibernation, leading to excessive fat metabolism and premature fat depletion. Understanding how insulin and the PI3K/Akt pathway are altered in WNS-affected bats could provide insights into why affected bats experience such drastic fat loss.

Insulin also plays a role in maintaining skeletal muscle by activating the PI3K/Akt signaling pathway. Upon activation, p-Akt influences skeletal muscle mass by regulating protein synthesis and degradation pathways. Specifically, phosphorylation by p-Akt leads to inactivation of FoxO3a, which prevents protein degradation and, thereby, muscle atrophy. Concurrently, activation of mTOR by p-Akt promotes muscle hypertrophy through protein synthesis [30,31]. In 13-lined ground squirrels (*Ictidomys tridecemlineatus*) p-Akt levels were reduced during hibernation. Interestingly, in the absence of p-Akt, serum- and glucocorticoid-inducible kinase 1 (SGK1) levels increased, along with a rise in p-FoxO3a [32]. SGK1 phosphorylated FoxO3a rendering it inactive and unable to promote muscle atrophy, while activation of mTOR further prevented atrophy [32]. In hibernating little brown bats, FoxO3a levels did not change either, but myostatin and histone deacetylase 4 (HDAC4) decreased along with an increase in SMAD7 [33]. Myostatin and HDAC7 inhibit muscle growth, maintenance and differentiation, while SMAD7 can counteract the inhibitory effects of myostatin on muscle growth and maintenance [34,35]. These studies highlight the importance of mTOR and FoxO3a in preventing muscle atrophy during prolonged inactivity. Dysregulation in the mTOR and FoxO signaling pathways, leading to muscle atrophy, could add stress for bats attempting to fight off the effects of WNS.

Insulin affects fat accumulation and prevention of muscle atrophy but also regulates immune functions via the PI3K/Akt pathway. In WNS-affected little brown bats, tumor necrosis factor alpha (TNFα), along with interleukin (IL)-10 and -23 were elevated in the lungs [36]. TNF plays a crucial role in initiating innate immune responses against fungal pathogens [37]. IL-23 is a pro-inflammatory cytokine and helps in the maturation of T helper cells. IL-10 is an inti-inflammatory cytokine synthesis inhibitory factor as it negatively regulates inflammatory responses and helps in B-cell survival and proliferation and antibody synthesis [36]. Expression of IL-6 is also increased in wing tissues of WNS-affected bats, which is likely related to the Th17 response, used to help clear out cutaneous fungal infection [38]. The PI3K/Akt/mTOR pathway is involved in the positive regulation of IL-23 [39] and IL-10 [40] in dendritic cells. The P13K/Akt pathway is also involved in the stimulation of TNF and activated NF-κB, which activates gene expression required for several immune responses [41]. Increasing evidence also points towards the role of this pathway in thymocyte survival and differentiation [42,43]. MicroRNAs (miRNAs) are small (20–25 nucleotides) non-coding RNAs. miRNAs regulate gene expression post-transcriptionally by inhibiting the translation or the stability of the target mRNA. The primary miRNA is transcribed from the DNA template by RNA polymerase (Pol II), which undergoes a maturation process by the action of the Microprocessor complex, constituted of RNase III enzyme Drosha and two DGCR8 proteins, creating shorter precursor miRNA (pre-miRNA) molecules. The pre-miRNA molecule is transported to the cytoplasm, where a mature miRNA is generated by the action of the Dicer enzyme [44]. The mature miRNA species may be derived from both the 5′ and 3′ strands of the precursor duplex, generating miRNA-5p and miRNA-3p. Both variants can be loaded into the Argonaute (AGO) family of proteins in an ATP-dependent manner [45]. The proportion of AGO loaded 5p or 3p strands varies greatly for any given miRNA, ranging from similar proportions to predominantly one or the other, with the cell type or cellular environment playing a significant role [46]. miRNAs have been widely used as indicators of disease in human health. They were first explored as a biomarker for leukemia [47], and are now widely used in cancer research. They can also provide insights into molecular mechanisms, progression and severity of infectious disease. miRNAs have been consistently detected during various infectious stages even when conventional biomarkers, like proteins and genetic mutations, have not been detected. In infectious diseases like HIV-1, changes in miRNA expression occur throughout the course of the disease, sometimes providing cues about the immune status of individuals [48,49]. Recently, miRNAs have also been implicated in diseases of livestock and wildlife diseases like Johne’s disease in domesticated cattle and the feline panleukopenia virus, where miRNA expression differentiates affected and unaffected populations [50,51]. Beyond functioning as biomarkers, miRNAs also regulate physiological processes important for hibernation. In brown bears (*Ursus arctos*), for example, miRNAs were involved in metabolic suppression and skeletal muscle maintenance during hibernation [52]. In hibernating little brown bats, one study identified eight miRNAs involved in neuroprotection [53] while another found that a different set of eight miRNAs helped prevent muscle atrophy [33].

Early in the outbreak of WNS, Iwanowicz et al. [54] explored the potential of miRNAs as biomarkers of *Pd* infection in little brown bats. They collected liver tissue from WNS-affected and unaffected bats and used next-generation sequencing to identify miRNAs in both groups. They found 167 miRNAs expressed in both WNS-affected and unaffected groups but identified 43 miRNAs that were only expressed in WNS-affected bats [54]. These 43 miRNAs could serve as potential biomarkers of *Pd* infection and WNS survival. A given miRNA typically influences multiple biological pathways by targeting genes involved in various processes [55,56,57]. Out of the 43 miRNAs identified by Iwanowicz et al. [54], we sought to identify a subset of miRNAs that could be most useful as WNS biomarkers if they regulate multiple pathways that are likely important for determining WNS severity and bat survival. Our first objective was, therefore, to identify miRNAs critical to WNS using bioinformatics tools to investigate roles of these 43 miRNAs in lipid storage and metabolism, insulin signaling as a driver of energy balance (including its roles in muscle growth and atrophy), and immune system regulation, pathways which likely influence bat hibernation, generally, and responses to *Pd* infection. Our second objective was to assess whether candidate miRNAs we identified using bioinformatics could be detected from wild-captured bats. Iwanowicz et al. [54] used samples of liver tissue from euthanized bats to test for miRNA expression, but less invasive samples would be preferable for studying endangered species. Therefore, as a proof-of-concept, we captured little brown bats from a WNS-affected population and used plasma samples to quantify the prevalence of candidate miRNAs identified through our in silico analysis. To our knowledge, no study has tested whether miRNA can be detected using the small plasma volumes that can be collected non-lethally from little brown bats. We report the first detections of miRNAs in plasma from *Myotis lucifugus* and the first prevalence data for four candidate miRNAs (i.e., miR-543-3p, miR-27a-5p, miR-92b-3p and miR-27a-3p) that could serve as biomarkers for WNS and the health status of hibernating little brown bats.

## 2. Results

### 2.1. Putative miRNA Targets Identified by Literature Review

We conducted a literature review and identified 18 of the 43 miRNAs uniquely expressed in WNS-affected bats, which regulate at least one of fat metabolism, insulin signaling or the immune system in mice (*Mus musculus*) and humans (*Homo sapiens*). Of these 18 miRNAs, we retained 6 (miR-92b, miR-27a, miR-543, miR-128-1, miR-10b and miR-328) that met our criterion of regulating at least two of our pathways of interest (Table 1). These six miRNAs were used for further enrichment analysis.

### 2.2. Pathway Enrichment Analysis of miRNA Targets Using Online Databases

**Figure 1:**
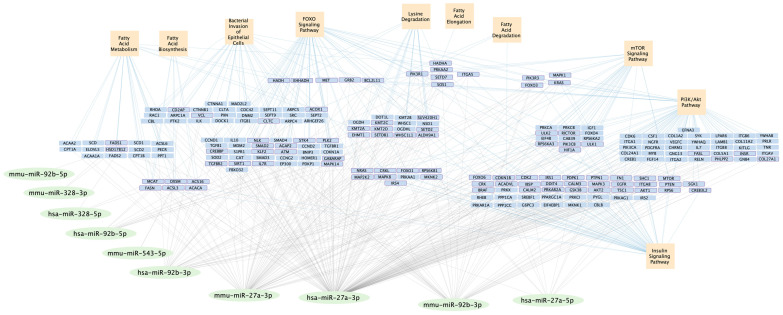
Cluster diagram showing the miRNA–target gene–pathway regulatory network, as predicted by our in silico analysis. miRNAs are represented by light green ellipses, target genes by light blue rectangles, and metabolic and immune response pathways by peach squares. The complex network illustrates how miRNAs regulate pathways through multiple genes, with crosstalk between pathways.

Our literature review suggested miR-128-1’s involvement in insulin and fatty acid metabolism [62,63] but the DIANA-miRPath analysis did not confirm its involvement in these pathways, so we did not analyze it further. Similarly, miR-10b-3p was not identified by DIANA-miRPath as regulating insulin and fatty acid metabolic pathways in mouse and human. miR-10b-5p was involved in fatty-acid biosynthesis in humans via regulation of the FASN gene but, because miR-10b was found to only regulate FASN and no other pathways of interest, we did not include it in subsequent analyses. We, therefore, proceeded with analyses of the remaining four miRNAs—miR-27a, miR-328, miR-92b and miR-543. miR-543 was not available in the DIANA-miRPath database for *Homo sapiens*, so human-pathway analysis included only miR-27a, miR-328, miR-92b and miR-10b. Further analysis with DIANA-miRPath revealed pathways regulated by the remaining four miRNAs in both mice (Table 2) and humans (Table 3). A network comprising 66 target genes regulated by selected miRNAs (mmu-miR-27a-3p, mmu-miR-328-3p, mmu-miR-92b-3p, mmu-miR-92b-5p, mmu-miR-543-5p, hsa-miR-27a-3p, hsa-miR-27a-5p, hsa-miR-328-5p, hsa-miR-92b-3p, hsa-miR-92b-5p) was constructed using Cytoscape 3.10.3 software to illustrate their involvement in key metabolic and signaling pathways. Among these, 28 genes (highlighted with a purple border in Figure 1) are regulated by multiple miRNAs and/or involved in multiple pathways (Figure 1).

### 2.3. miRNAs Involved in Fat Metabolism

The analysis using DIANA-miRPath showed that regulation of fatty acid biosynthesis, metabolism, elongation and degradation involves a complex interplay of multiple miRNAs.

In mice, fatty acid biosynthesis was linked to miR-27a-3p and miR-543-5p by FASN, OXSM and ACS16 genes (*p* < 0.001). Fatty acid metabolism in mice was regulated by miR-27a-3p, miR-328-3p, miR-92b-5p and miR-543-5p by targeting 13 genes (*p* < 0.001). Biosynthesis of unsaturated fatty acids was regulated by 2 genes linked to miR-328-3p (*p* = 0.04) and fatty acid elongation was regulated by miR-27a-3p in mouse (*p* < 0.001) (Table 2 and Table S1).

In humans, the fatty acid metabolism pathway was associated with miR-27a-3p, miR-27a-5p, miR-328-5p, miR-92b-5p and hsa-miR-10b-5p via 14 genes (*p* < 0.001) (Table 3). miR-27a-5p and miR-92b-5p were found to regulate fatty acid degradation by targeting three genes (*p* = 0.001) (Table 3 and Table S1). The lysine degradation pathway, which is involved in fat metabolism, was regulated by miR-27a-3p in mice and miR-27a-3p and miR-92b-3p in humans via six genes (*p* < 0.0001).

### 2.4. miRNAs Involved in Insulin Metabolism

The DIANA-miRPath analysis showed that the insulin signaling pathway was regulated by miRNA-27a-3p, miR-27a-5p and miR-92b-3p in humans via its influence on 61 genes (*p* < 0.05) (Table 3 and Table S2). The PI3K/Akt pathway was regulated by miR-92b-3p in mice (*p* < 0.05) by influencing 19 genes (Table 2 and Table S2).

### 2.5. miRNAs Involved in Skeletal Muscle Maintenance

We found that the FoxO signaling pathway was regulated by miR-27a-3p and miR-92b-3p in humans (*p* < 0.05) and mice (*p* < 0.001) suggesting a role in skeletal muscle maintenance.

### 2.6. miRNAs Involved in Immune System Regulation

The enrichment analysis revealed that in humans, the bacterial invasion of epithelial cells KEGG pathway was regulated by miR-27a-3p and miR-92b-3p, involving 41 genes (*p* < 0.001) (Table 3 and Table S4). Our in-depth analysis of the four miRNAs revealed significant and overlapping roles of four miRNAs—miR-543-5p, miR-27a-3p, miR-92b-3p and miR-328-5p that could be relevant to WNS progression and survival.

### 2.7. Prevalence of miRNAs in Blood Plasma of Wild Bats

**Figure 2:**
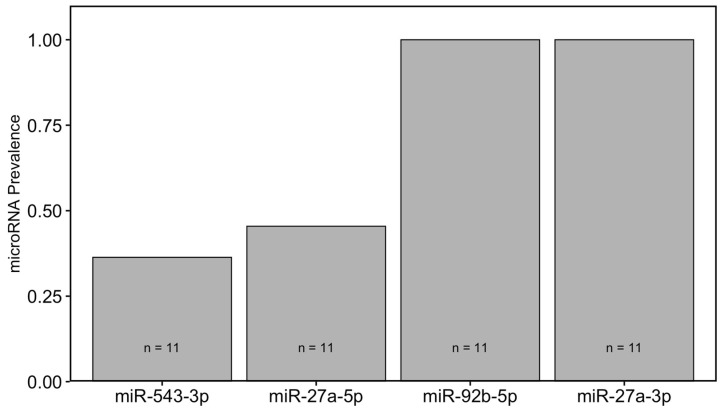
Prevalence of the selected miRNAs (miR-543-3p, miR-27a-5p, miR-92b-5p and miR-27a-3p) in plasma samples collected from little brown bats during fall fattening.

We obtained blood plasma samples from 11 little brown bats (adult female: 2, adult male: 9, young-of-the-year male: 1; body mass = 11.45 ± 1.42) to determine whether the miRNAs identified by in silico analysis (i.e., miR-543-3p, miR-27a-5p, miR-27-3p and miR-92b-3p) could be detected in plasma. Prevalence varied across miRNAs, with 100% prevalence for miR-92b-5p and miR-27a-3p (11/11; 2/2 F, 9/9 M). miR-27a-5p was detected in 45% of individuals (11/11; 2/2 F, 3/9 M) and miR-543-3p had the lowest prevalence at 36% (4/11; 0/2 F, 4/9 M; Figure 2).

## 3. Discussion

Based on our literature review, of the 43 miRNAs expressed in WNS positive bats [54] are of particular importance because each one influences multiple pathways, including insulin signaling, that may affect WNS pathophysiology and bat survival. Based on our in silico analyses, we narrowed down 43 miRNAs exclusively expressed in WNS affected bats [54] to four miRNAs. Due to the limited information available on bat miRNAs, we used mouse and human miRNA data to predict the metabolic and immune response pathways influenced by these selected miRNAs. We constructed a miRNA-gene-pathway cluster diagram (Figure 1) showing the potential regulatory effects of miRNAs on pathways that could be involved in the pathogenesis of WNS [16,18,20,21,22,23].

Iwanowicz et al. [54] reported the presence of miR-27a-5p in WNS-affected little brown bats. We found that miR-27a-5p’s influence is limited to fatty acid metabolism, degradation and elongation, and insulin signaling pathway. Xie at al. [89] highlighted a negative role for miR-27a-5p, particularly in its ability to inhibit SREBP-1c. SREBP-1c is crucial for adipocyte differentiation and lipogenesis, so its downregulation could negatively impact a bats’ ability to fight off WNS infection. The visceral adipose secreting extracellular vesicle—containing miR-27a-3p targets pancreatic β-cells and impairs insulin secretion by inhibiting CACNA1c [90]. Our analysis did not find CACNA1c and SREBP-1c as target genes of miR-27a-5p. Although miR-27a-5p is uniquely expressed in WNS-positive bats [54] and is involved in fatty acid metabolism and insulin signaling, we detected this miRNA in only 45% of our samples from wild bats. This suggests it may play a less fundamental regulatory role, at least during the fall fattening phase. Recent research on miRNAs has confirmed that both -3p and -5p variants can co-exist [91]. These variants may regulate similar or distinct pathways depending on tissue context and disease state [91,92]. Consistent with this, previous studies have found that miR-27a-5p and -3p variants can co-exist, and exhibit tissue-specific expression and distinct regulatory roles in various solid tumors [93,94].

Our literature review revealed a strong negative correlation between miR-27a-3p and the PI3K/Akt pathway, with miR-27a-3p modulating GLUT4 via direct targeting of PPARγ, an activator of the pathway [95,96,97,98]. miR-27a-3p also negatively targets various adipogenic markers like FASN [99,100], PLIN1 [67] and SCD1 [101,102]. Wu et al. reported that miR-27a-3p is crucial regulator for adipogenesis, not miR-27a-5p, by targeting PPAR-γ, despite both miR-27a-5p and miR27a-3p were downregulated during the adipogenic differentiation [103]. Meanwhile, expression of CPT1B, involved in the breakdown of fatty acids [104], increased with an increase in miR-27a-3p [105]. Our in silico analysis revealed that miR-27a-3p targets CPT1A/B, SCD1 and FASN (Table S1). Given that SCD1 and FASN are key enzymes for lipogenesis, whereas CPT1 is essential for fatty acid breakdown, these findings suggest that miR-27a-3p reduces overall fat accumulation by upregulating CPT1B (thereby enhancing fatty acid oxidation) and inhibiting FASN and SCD1 (thus reducing lipogenesis).

When insulin binds to its receptor, IRS1 becomes activated and triggers the PI3K/Akt pathway. Because miR-27a-3p downregulates PI3K/Akt signaling, a corresponding decrease in IRS1 levels is expected. Indeed, IRS1 is negatively regulated by miR-27a-3p [60]. Given IRS1’s central role in PI3K/Akt signaling, it is unsurprising that our analysis identified IRS1 as a miR-27a-3p target involved in insulin signaling, as well as pathways regulated by PI3K/Akt, including mTOR signaling, FoxO signaling, and B-cell and T-cell receptor pathways. miR-27a-3p may influence mTOR and FoxO signaling in WNS-affected bats by modulating the PI3K/Akt pathway. miR-27a-3p has been shown to increase mTOR expression by negatively regulating SYK in humans [106]. It also downregulates TSC1, activating the mTOR signaling pathway [107,108], which could help prevent muscle atrophy. Conversely, miR-27a-3p inhibits the expression of FOXO1 [109]. Our in silico analysis confirmed SYK, TSC1 and FOXO1 as targets of miR-27a-3p (Tables S2–S4).

In contrast to miR-27a-5p, miR-27a-3p showed a broader regulatory impact and was consistently expressed in all plasma samples we tested. Our in silico analysis identified several pathways influenced by miR-27a-3p in mice and humans, including immune response modulation, cellular stress adaptation, and metabolic processes, which could affect progression of WNS. Although miR-27a-3p was not expressed in liver tissue samples collected from WNS-affected little brown bats, we included it in our analysis based on its predicted involvement in multiple WNS-relevant pathways. Key predicted targets include IRS1, SYK, TSC1, and FOXO1, suggesting that miR-27a-3p may influence metabolism and immune system regulation during hibernation. While it may not be exclusive to WNS, its consistent presence in all plasma samples tested (Figure 2) suggests that miR-27a-3p plays a central role in regulating metabolic pathways in little brown bats. Through its involvement in lipid metabolism, insulin signaling, and immune system pathways, it may contribute to physiological adaptations essential for overwinter survival.

miR-328-3p exerts an inhibitory effect on the PI3K/Akt pathway [110] and on fatty acid oxidation by downregulating CPT1A [111] but our in silico analysis only identified involvement of this miRNA in fatty acid metabolism and biosynthesis of fatty acids via regulation of FADS1 and HSD17B12 (Table S1). The inhibitory effect of miR-328-3p on the PI3K/Akt pathway is further illustrated by its positive regulation of PTEN, which inhibits the P13K/Akt pathway [112]. miR-328-3p also inhibits this pathway by targeting ITGA5 [113]. Our analysis pointed to the role of miR-328-5p in metabolism and biosynthesis of fatty acids by regulating FADS2, FASN and MCAT (Table S1). While there is no evidence of regulation of MCAT or FADS2 by miR-328-5p in the literature, it has been shown to negatively regulate adipogenesis by targeting FASN and PPAR-γ [114]. Unfortunately, primers were not available for this miRNA, and we could not confirm its prevalence in our population of WNS-affected little brown bats, but it would be worthwhile to test for given its role in fatty acid metabolism, fatty acid elongation and insulin secretion.

miR-543-5p was also present in WNS-affected little brown bats [54]. Hu et al. [88] demonstrated the role of miR-543 in inhibiting SIRT1 and glycogen synthase which disrupted the PI3K/Akt pathway and glycogen synthesis. SIRT1 is also directly linked to lipolysis with lowered PPARγ expression [115]. Our DIANA analysis suggested the role of miR-543-5p in fatty acid metabolism and biosynthesis via regulation of OXSM and FASN, respectively, we found no evidence of this in our literature review. We could not test for the prevalence of miR-543-5p in wild-captured bats but we did detect miR-543-3p in some bats. Prevalence of this miRNA was relatively low (36%), however, and our in silico analysis revealed no relationship between this miRNA and any of our pathways of interest which suggests it could be a lower priority for future studies of the functional role of miRNAs in bat hibernation and WNS.

Yang et al. [116] highlighted the detrimental role of miR-92b-3p in skeletal muscle function because miR-92b-3p promotes expression of Atrogin-1 and MuRF-1 (key markers of muscle degradation) and suppresses pathways that support muscle maintenance, such as UGP2 and mTOR signaling. In another study, miR-92b-3p activated the mTOR signaling pathway by targeting and downregulating TSC1, a known negative regulator of mTOR [117]. In a third study, this miRNA was also involved in upregulating p-Akt, downregulating PTEN and thereby activating the PI3K/Akt pathway [118]. Based on our analysis, miR-92b-3p regulates the insulin signaling pathway, which includes PI3K, Akt, mTOR, and PTEN as key components. The FOXO signaling pathway also emerged as a pathway enriched by miR-92b-3p. A recent study found that FOXO1 suppresses miR-92b expression, indirectly influencing downstream stress-response genes [119]. Since FOXO1 is inhibited by PI3K/Akt signaling, miR-92b-3p likely interacts with the insulin signaling pathway, a pathway that activates PI3K/Akt, leading to modulation of downstream targets like mTOR and FOXO.

Iwanowicz et al. [54] showed that miR-92b-5p, was present in WNS-affected but not unaffected little brown bats. Yang et al. [116] suggested miR-92b-5p could be involved in muscle fatigue by targeting MCT4 and aiding lactate buildup. However, we did not find MCT4 as a predicted target of miR-92b-5p. Instead, fatty acid metabolism was associated with miR-92b-5p in our enrichment analysis but with no evidence of these functions found in the literature. The literature highlighted the role of miR-92b-5p in suppressing IL-18 [120] but we found no evidence of it in the DIANA-miRPath database. We tested the prevalence of miR-92b-5p and detected it in all samples tested.

Our study narrowed down the 43 miRNAs identified by Iwanowicz et al. [54] as those associated with WNS-affected bats to four candidates (miR-27a, miR-92b, miR-328, and miR-543) that we hypothesize may play disproportionate roles in WNS pathophysiology and bat survival due to their involvement in pathways likely important for WNS. These miRNAs exhibit differences in their regulatory roles in mice and humans, and our in silico analysis helps characterize those differences. While miR-27a-3p, miR-92b-3p, and miR-543-5p showed involvement in multiple signaling pathways related to fat metabolism, the insulin pathway and immune modulation, miR-27a-5p and miR-92b-5p appeared to have more limited regulatory roles. Although we found some studies suggesting roles for miR-328 and miR-543, these could not be corroborated by our pathway enrichment analysis.

We quantified prevalence of miRNAs highlighted by our in silico analysis in plasma samples collected from little brown bats persisting after WNS invasion. This study represents what is, to our knowledge, the first detection of miRNAs in plasma samples from wild-captured little brown bats which adds to the limited knowledge of miRNA profiles in wildlife. Non-lethal sampling methods are particularly important for research on endangered species so confirming miRNA prevalence in plasma helps set the stage for future studies of little brown bats and other wildlife species. Of the miRNAs we assayed, prevalence of miR-543-3p was lowest. Iwanowicz et al. [54], detected miR-543-5p—the strand for which we were unable to test because the corresponding primer was not available. The low detection of miR-543-3p may indicate that this strand is less relevant compared to miR-543-5p, the miRNA assayed by Iwanowicz et al. [54] or could reflect a limited role for this miRNA in WNS and/or preparation for hibernation. Additional studies would be useful to better understand functions of these two sequences of miR-543. Prevalence of miR-27a-5p was higher than miR-543-3p but also varied among individuals, possibly indicating differential expression that could provide insights into the pathophysiology of WNS or hibernation physiology. miR-27a-3p and miR-92b-5p were detected in all samples we tested. The consistent presence of these miRNAs suggests they could play fundamental roles in metabolic adaptations relevant to WNS and/or hibernation. Future studies correlating miRNA expression levels with disease severity or metabolic status could clarify their functional importance and potential as biomarkers.

The observed discrepancies between our bioinformatics predictions and the existing literature can be attributed to several factors. Firstly, pathway enrichment tools rely on algorithms to predict gene targets, which are important for preliminary studies to help narrow down potential miRNAs for subsequent experimental validation. Secondly, databases such as TarBase, despite using experimental evidence, may not include the latest findings. These limitations underscore the importance of complementing bioinformatic analyses with laboratory validation. Our results could help guide future studies aimed at understanding the role of miRNAs in the physiology of hibernating bats in general, as well as their roles in WNS. Our study confirmed several miRNAs that were consistently detected across plasma samples, suggesting their stable presence in plasma and potential as non-invasive biomarkers. Confirming the abundance of miRNAs in plasma samples of little brown bats should enable more targeted research to understand the functions of these miRNAs. Future studies investigating the expression of these miRNAs should test for correlations between body condition of bats or disease severity and miRNA expression and attempt to validate the gene targets of these miRNAs to corroborate our in silico findings.

## 4. Materials and Methods

### 4.1. Literature Search Strategy

We searched the literature for studies on the 43 miRNAs differentially expressed in WNS-affected bats [54] using Pubmed, Google Scholar and PubMed Central. We used unique searches for the names of each of the 43 miRNAs along with “Insulin Signaling Pathway,” “Immune System Regulation,” and “Lipid Metabolism” as search terms. Studies we found relied on a range of tissue samples, including serum, plasma, urine and saliva. Since there is little information on miRNAs involved in wildlife diseases, we had to rely on studies of humans and mice. Only those miRNAs found in at least two pathways were selected for pathway enrichment analysis to ensure we focused on the most important miRNAs.

### 4.2. Pathway Enrichment Analysis of miRNA Targets

We conducted pathway enrichment analysis using DIANA-miRPath v3.0 “http://www.microrna.gr/miRPathv3, (accessed on 20 February 2025)” [121,122] to validate findings from the literature review and to identify miRNA targets associated with key biological pathways. For this study, predicted miRNA targets were retrieved for *Homo sapiens* and *Mus musculus*. This choice was based on three factors: most studies in our literature review were conducted in human or mouse models; the DIANA-miRPath database does not include *Myotis* species, and mature miRNA sequences are highly conserved across mammals, allowing for reasonable cross-species inference. Additionally, since the original study by Iwanowicz et al. [54] did not specify the strand (-3p or -5p) for each miRNA, we included both -3p and -5p strands in our analysis where applicable. This allowed us to ensure that potentially relevant strand-specific targets were not excluded.

DIANA-miRPath integrates three resources for miRNA interaction and pathway analysis: DIANA-microT-CDS uses a trained algorithm to predict miRNA-gene interactions [123], TargetScan 6.2 also predicts miRNA-gene interactions [124], and DIANA-TarBase v7.0 validates predicted interactions using experimentally confirmed evidence [125]. These predicted and validated targets were then mapped to biological pathways using the Kyoto Encyclopedia of Genes and Genomes (KEGG) database, enabling identification of the pathways regulated by the selected miRNAs.

Given the exploratory nature of our study, we used the “Pathway Union” setting in DIANA-miRPath to identify all significantly targeted pathways by the selected miRNAs. This setting performs an initial enrichment analysis to calculate *p*-values for the association between each miRNA and every pathway. These individual significance levels are then combined using Fisher’s exact method to calculate a merged *p*-value for each pathway. The merged *p*-value represents the probability that the pathway is significantly enriched with gene targets of at least one selected miRNA. This approach ensures all pathways targeted by multiple miRNAs are included, enabling a broader exploration of potential miRNA-pathway interactions. Pathways with a merged *p*-value of less than 0.05 were considered statistically significant. The miRNA–target gene–pathway regulatory network was constructed and visualized using Cytoscape 3.10.3 software (https://cytoscape.org/, accessed on 14 May 2025).

### 4.3. Study Site and Animal Collection

We captured bats for plasma sampling using a harp trap placed at the entrance of St. George Bat Cave in the Lake St. George Caves Ecological Reserve ~30 km north of Fisher River Cree Nation, Manitoba (51°72′ N 97°41′ W) between 6 September and 29 September 2022, prior to hibernation. This little brown bat hibernaculum housed approximately 10,000 bats each winter prior to WNS. It has been WNS-positive since 2017–2018 and housed about 2500 bats as of March 2024.

All procedures were conducted under Manitoba Natural Resources and Northern Development Species at Risk/Wildlife Scientific (SAR21013) and Ecological Reserves permits. We closely followed Canadian Wildlife Health Cooperative and U.S. Fish and Wildlife Service guidelines for decontamination of *Pseudogymnoascus destructans* [126,127].

We held bats in individually marked brown paper bags until processing. We recorded sex, mass (±0.1 g) and age {adult (>1 year) or young-of-the-year (<1 year)} based on ossification of the metacarpal-phalangeal joints [128,129] for each individual. After plasma sampling (see below) all bats were implanted with a passive transponder (PIT tag) so we could avoid double sampling from any recaptured bats and then released at the site of capture.

### 4.4. Plasma Sampling

We collected a maximum of 6 µL of blood per gram of body mass [130] from little brown bats captured in the fall. We cleaned the venipuncture site in the interfemoral region with an alcohol swab. Then, we punctured the interfemoral vein with the tip of a 27G × 1/2-inch syringe needle (Becton, Dickinson and Company, Mississauga, ON, Canada) and collected approximately 70 µL of blood in a heparinized capillary tube (Fisher Scientific, Pittsburgh, PA, USA) [19,131]. We sealed the one end of the capillary tubes using Critoseal or Critocaps (McCormick Scientific, St. Louis, MO, USA). We centrifuged the blood within an hour of collection at 10,000× *g* for 5 min in a hematocrit centrifuge (Zipocrit™; LW Scientific Inc., Lawrenceville, GA, USA) and transferred plasma to 1 mL cryovials (Fisherbrand; Thermo Fisher Scientific, Pittsburgh, PA, USA). We stored the cryovials in a cryoshipper (Taylor-Wharton, Theodore, AL, USA) filled with liquid nitrogen until transportation to the University of Winnipeg [19].

### 4.5. RNA Extraction and cDNA Synthesis Using Plasma Samples

We extracted RNA from plasma using the miRNeasy serum/plasma kit (Cat. #217204, Qiagen, Venlo, The Netherlands) following the manufacturer protocol with a few modifications. We checked the RNA concentration and purity using a microplate spectrophotometer (SpectraMax i3, Molecular Devices, San Jose, CA, USA) by examining the ratio of absorbance at 230, 260 nm and 280 nm.

A highly specific cDNA reverse transcription for miRNA requires 5 µL of RNA (1–10 ng). Therefore, we diluted the extracted RNA using nuclease-free water to contain ~7 ng of RNA, which was then used for cDNA synthesis. We synthesized cDNA using the TaqMan™ MicroRNA Reverse Transcription Kit (Cat. #4366596, ThermoFisher Scientific, Mississauga, ON, Canada) following the manufacturer protocol

### 4.6. Identification of Homologous Sequence for RT-qPCR Primers

Following our in silico analysis, we identified four miRNAs (miR-92b, miR-27a, miR-543 and miR-328) whose 5p and/or 3p strands were predicted to regulate two or more pathways of interest. To select the appropriate primers for RT-qPCR, we used miRBase v.22 (http://www.mirbase.org/) to retrieve the exact mature miRNA sequences for those reported by Iwanowicz et al. [54] (Table 4). The ‘Search by Sequence’ function of miRBase uses RNAcentral’s comprehensive miRNA database to compare our *M. lucifugus* miRNA nucleotide sequences to identify miRNAs in other species [132,133].

Where possible, we matched these sequences to available assay primers to ensure consistency between our qPCR assays and the miRNAs originally identified. miR-27a-3p was not reported by Iwanowicz et al. [54] in WNS-positive bats but we included it in our analysis due to our in silico results showing its involvement in multiple WNS-related pathways. miR-27a-5p, on the other hand, was reported by Iwanowicz et al. [54] in WNS-positive bats and we also included this sequence based on its predicted relevance in our pathway analysis. miR-92b-3p was not reported by Iwanowicz et al. [54] and showed a limited role in relevant pathways but miR-92b-5p was reported by Iwanowicz et al. [54] and was important to WNS-relevant pathways so we included the 5p variant of this miRNA. An exact primer sequence match to the miR-92b-5p reported by Iwanowicz et al. [54] was unavailable from ThermoFisher so we used BLAST v. 2.12.0 analysis to identify a primer with 95% sequence similarity to the miR-92b-5p sequence from Iwanowicz et al. [54] and used this primer for our RT-qPCR assays (Table 5).

In silico analysis for miR-543-3p and miR-543-5p did not reveal their strong role in multiple pathways, but we still included these sequences based on multiple studies reporting their role in the insulin signaling pathway. The primer for miR-543-5p was unavailable from ThermoFisher, and despite being reported by Iwanowicz et al. [54], we could not test this miRNA. Instead, we tested for the presence of miR-543-3p in our samples. miR-328-5p was reported by Iwanowicz et al. [54] and showed strong evidence of involvement in multiple pathways in our analysis, but the sequence for this miRNA was also unavailable, so we also excluded it from our analysis. We excluded miR-328-3p due to no evidence of its involvement in WNS-related pathways.

The DIANA-miRPath library did not contain miRNAs specific to *M. lucifugus*. By aligning the sequences of the six miRNAs to the miRBase v.22 database, we identified human and mouse miRNAs as the closest matches, (Table 2), and these were subsequently used for further analysis. The miRNA output sequences by miRBase aligned fully with human or mouse miRNA sequences, and the sequence alignment is provided as supplementary data (Table S5).

### 4.7. Reverse Transcription Quantitative Real-Time Polymerase Chain Reaction (RT-qPCR)

We performed RT-qPCR by adding 0.67 µL of cDNA to 0.5 µL of TaqMan™ Small RNA Assay (20×) (Cat. #4427975, ThermoFisher Scientific, ON, Canada) and 5.0 µL of TaqMan™ Fast Advanced Master Mix (Cat. #4444558, ThermoFisher Scientific, ON, Canada) and 3.84 µL nuclease-free water in a final volume of 10 µL. The amplification and detection were performed in a 96-well plate and CFX Connect Real-Time PCR Detection System (Bio-Rad, Toronto, ON, Canada). The thermal profile that we used is as follows: 50 °C for 2 min, 95 °C for 20 s, 40 cycles of 95°C for 1 s, and 60 °C for 20 s. Each sample was run in triplicates to ensure accuracy and reliability, with a cut-off set to 40 cycles. We used primers for hsa-miR-27a-5p, mmu-miR-92b-5p, mmu-miR-543-3p and hsa-miR-27a-3p (Table 5; Cat. # 4427975, ThermoFisher Scientific, ON, Canada).

## Figures and Tables

**Table 1:** Functional roles of 43 miRNAs, unique to WNS-positive little brown bats, in biological pathways relevant to WNS, including insulin signaling, lipid metabolism, and immune system regulation. Numbers in brackets [ ] indicate references from the literature review that support the roles of miRNAs in biological systems. miRNA in bold are present in two or more pathways and were selected for the in silico analysis.

miRNAs	Immune System Regulation	Lipid Metabolism	Insulin Signaling Pathway
**miR-101c**	No	No	No
**miR-10b**	Yes [58,59]	Yes [60]	Yes [61]
**miR-28b**	No	No	No
**miR-128-1**	No	Yes [62,63]	Yes [63]
**miR-133a-1//miR-133a-2**	Yes [64]	No	No
**miR-135b**	No	No	No
**miR-181d**	No	No	No
**miR-202**	No	No	No
**miR-217**	No	Yes [65,66]	No
**miR-302a**	No	No	No
**miR-328**	Yes [67]	No	Yes [68]
**miR-342**	No	No	Yes [69]
**miR-369**	Yes [70]	No	No
**miR-377**	No	No	Yes [71]
**miR-425**	No	Yes [72]	No
**miR-483**	No	No	Yes [73,74]
**miR-501**	No	No	No
**miR-675**	No	No	No
**miR-1306**	No	No	No
**miR-1957**	No	No	No
**miR-3967**	No	No	No
**miR-27a**	Yes [75]	Yes [76,77]	Yes [78]
**miR-92b**	Yes [79]	No	Yes [80]
**miR-132**	No	No	No
**miR-135a-1//miR-135a-2**	No	No	No
**miR-154**	No	No	No
**miR-1843b**	No	No	No
**miR-203**	Yes [81]	No	No
**miR-219-1**	No	No	No
**miR-3096**	No	No	No
**miR-340**	No	No	No
**miR-350**	Yes [82]	No	No
**miR-375**	No	No	Yes [83]
**miR-412**	No	No	No
**miR-450a-1//miR-450a-2**	No	No	No
**miR-495**	No	No	Yes [84]
**miR-539**	No	No	No
**miR-544**	Yes [85]	No	No
**miR-758**	No	No	No
**miR-1895**	No	No	No
**miR-3966**	No	No	No
**miR-5112**	Yes [86]	No	No
**miR-543**	No	Yes [87]	Yes [88]

**Table 2:** Roles, and numbers of genes regulated, for the miRNAs we studied in various pathways in *Mus musculus* identified using DIANA-miRPath version 3.0 software.

Pathway	*p*-Value	Number of Genes Regulated by							
		mmu-miR-27a-3p	mmu-miR-27a-5p	mmu-miR-328-3p	mmu-miR-328-5p	mmu-miR-92b-3p	mmu-miR-92b-5p	mmu-miR-543-3p	mmu-miR-543-5p
Fatty acid biosynthesis	<0.001	2							1
Fatty acid metabolism	<0.001	10		2			1		1
Fatty acid elongation	<0.001	1							
FoxO signaling pathway	<0.001	31				21			
Lysine degradation	<0.001	13							
Biosynthesis of unsaturated fatty acids	<0.01			2					
PI3K-Akt signaling pathway	0.02					19			
T-cell receptor signaling pathway	0.31	30							
Fc gamma R-mediated phagocytosis	0.31	24				9			
Bacterial invasion of epithelial cells	0.35	22				8			
Fatty acid degradation	0.45	1							
mTOR signaling pathway	0.62	18							
B-cell receptor signaling pathway	0.76	18							

**Table 3:** Roles, and numbers of genes regulated, for the miRNAs we studied in various pathways in for *Homo sapiens* identified using DIANA-miRPath software. miR-543 was not available in the human database so is not included in this table.

Pathway	*p*-Value	Number of Genes Regulated by						
		hsa-miR-27a-3p	hsa-miR-27a-5p	hsa-miR-328-3p	hsa-miR-328-5p	hsa-miR-92b-3p	hsa-miR-92b-5p	hsa-miR-10b-5p
Fatty acid metabolism	<0.001	8	3		3		1	1
Lysine degradation	<0.001	14				8		
Fatty acid degradation	<0.001		2				1	
Bacterial invasion of epithelial cells	<0.001	31				10		
FoxO signaling pathway	<0.01	38				19		
Insulin signaling pathway	0.006	41	9			11		
Fatty acid biosynthesis	0.00	3			2			1
mTOR signaling	0.39	22						
Fatty acid elongation	0.09		1					
Insulin secretion	0.92				14			
PI3K-Akt signaling pathway	0.93	48						

**Table 4:** Sequence homology of miRNAs in *Myotis lucifugus* predicted by miRbase. The first column in the table lists sequences reported by Iwanowicz et al. [54] obtained by RNA-Seq and the second column lists the miRNA sequence output by miRbase that identified human and mouse miRNAs listed in column 3.

Sequence Reported in *Myotis lucifugus*	Sequence by miRBase	miRNA
TACCCTGTAGAACCGAATTTGTG	UACCCUGUAGAACCGAAUUUGUG	hsa-miR-10b-5p
AGGGCTTAGCTGCTTGTGAGCA	AGGGCUUAGCUGCUUGUGAGCA	hsa-miR-27a-5p
AGGGACGGGACGTGGTGCAGTGTT	AGGGACGGGACGUGGUGCAGUGUU	mmu-miR-92b-5p
GGGGGGCAGGAGGGGCTCAGGG	GGGGGGCAGGAGGGGCUCAGGG	mmu-miR-328-5p
AAGTTGCCCGCGTGTTTTTCG	AAGUUGCCCGCGUGUUUUUCG	mmu-miR-543-5p
CGGGGCCGTAGCACTGTCTGA	CGGGGCCGUAGCACUGUCUGA	mmu-miR-128-1-5p

**Table 5:** List of all the primers (miR-543-3p, miR-27a-5p, miR-92b-5p and miR-27a-3p) used for RT-qPCR along with their sequences.

Sequence Used for Primer Design	Target miRNA
AAACAUUCGCGGUGCACUUCUU	mmu-miR-543-3p
AGGGCUUAGCUGCUUGUGAGCA	hsa-miR-27a-5p
AGGGACGGGACGCGGUGCAGUG	hsa-miR-92b-5p
UUCACAGUGGCUAAGUUCCGC	hsa-miR-27a-3p

## Data Availability

The data used to support the findings of this study are available from the corresponding author upon request.

## Supplementary Materials

The supporting information can be downloaded at: https://www.mdpi.com/article/10.3390/ijms26178200/s1.
