# Supplementary material for: In Silico Analysis of Possible microRNAs Involved in the Pathogenesis of White-Nose Syndrome in Myotis lucifugus
Source: Int J Mol Sci. 2025 Aug 23;26(17):8200. doi: 10.3390/ijms26178200 (PMC12427802; doi:10.3390/ijms26178200)
Supplement: Supplementary file 1 [file ijms-26-08200-s001.zip › ijms-3769623-supplementary.pdf]

Supplementary Materials:

Table S1: miRNA-Targeted Genes in involved in pathways related to fat metabolism

Pathways	mmu-miR-27a-3p	mmu-miR-328-3p	mmu-miR-92b-5p	mmu-miR-543-5p	hsa-miR-27a-3p	hsa-miR-27a-5p	hsa-miR-328-5p	hsa-miR-92b-3p	hsa-miR-92b-5p
Fatty Acid Metabolism	FADS1	FADS1	ACAA1A	OXSM	HADH	HADHA	FADS2		ACOX1
	FASN	HSD17B12			FASN	ACADVL	FASN		
	SCD1				ACSL3	SCD	MCAT		
	SCD2				PPT1				
	ELOVL5				ELOVL5				
	ACAA2				CPT1B				
	EHHADH				HSD17B12				
	CPT1A				ACACA				
	PECR								
	ACSL6								
Fatty Acid Biosynthesis	FASN			ACSL3	FASN		FASN		FASN
	ACS16				ACSL3		MCAT		
					ACACA				
Fatty Acid Elongation	HADHA					HADHA			
Fatty Acid Degradation	HADHA					HADHA			ACOX1
						ACADVL			
Lysine Degradation	ALDH9A1				ALDH9A1			ALDH9A1	
	SETD2				SETD2			SETD2	
	SUV420H1				SUV420H1			SUV420H1	
	SETD7				SETD7			SETD7	
	WHSC1L1				WHSC1L1			WHSC1L1	
	KMT2C				KMT2C			KMT2C	
	DOT1L				DOT1L			HADH	
	EHMT1				EHMT1			KMT2A	
	KMT2D				KMT2D			SETDB1	
	OGDHL				OGDH				

NSD1				KMT2B				
EHHADH				HADH				
WHSC1				KMT2A				
				SETDB1				

Table S2: miRNA-Targeted Genes in involved in pathways related to insulin pathway

**Insulin Signaling Pathway**			**PI3K/Akt Pathway**	
**hsa-miR-27a-3p**	**hsa-miR-27a-5p**	**hsa-miR-92b-3p**	**mmu-miR-92b-3p**	**hsa-miR-27a-3p**
TSC1	CALM3	BRAF	PRKAA2	PRKAA2
PDPK1	SHC1	GSK3B	TSC1	TSC1
PIK3R3	PTPN1	NRAS	RPS6KB1	RPS6KB1
CALM3	RPS6	CRKL	BCL2L11	BCL2L11
FASN	CALM2	CRK	ITGA5	ITGA5
CBLB	AKT1	KRAS	ITGA8	ITGA8
RPS6KB1	MAPK3	AKT2	CREB3L2	PHLPP2
MKNK2	MKNK2	IRS4	IBSP	PRLR
GRB2	GRB2	PRKAR2A	PIK3R3	GSK3B
IRS1		MAPK1	DDIT4	PDGFRA
BRAF		IRS2	FASL	GNG13
PPP1CC			COL27A1	MET
PPP1CA			PHLPP2	MYB
GSK3B			PIK3CB	ITGB8
PYGL			ITGAV	LAMB1
CBL			COL1A2	FGF14
NRAS			SGK1	ITGA8
MAP2K2			PTEN	SYK

CRK			COL5A1	COL24A1
SHC1				ITGB6
PIK3CB				IL7
PRKCI				YWHAB
PTPN1				EFNA3
EIF4EBP1				EGFR
RHEB				ITGA1
SREBF1				KRAS
G6PC3				CDK6
MAPK8				LPAR6
PRKAG1				YWHAQ
PIK3R1				CREB1
PRKAR2A				RELN
PRKX				VEGFC
MAPK3				ITGA2
PRKAA1				SOS1
PRKAR1A				KITLG
PPARGC1A				COL11A2
MKNK1				IRS1
MTOR				INSR
FOXO1				CREB3L2
MAPK1				PIK3CA
ACACA				FN1
				TNR
				PDPK1
				CSF1
				GRB2
				GNB4
				NGFR
				CHRM1

Table S3: miRNA-Targeted Genes in involved in pathways related to protein metabolism

**mTOR Signaling Pathway**		**FoxO Signaling Pathway**			
**mmu-miR-27a-3p**	**hsa-miR-27a-3p**	**mmu-miR-27a-3p**	**mmu-miR-92b-3p**	**hsa-miR-27a-3p**	**hsa-miR-92b-3p**
BRAF	BRAF	BRAF	*BRAF*	BRAF	BRAF
HIF1A	HIF1A	MAPK14	*KRAS*	TGFBR1	TGFBR1
IRS1	IRS1	PIK3R1	*PIK3CB*	PIK3CB	KRAS
RPS6KB1	RPS6KB1	PDPK1	*BCL2L11*	NRAS	NRAS
PDPK1	PDPK1	PIK3R3	*FOXO1*	FOXO1	NLK
MTOR	MTOR	FBXO32	*MAPK14*	SETD7	SETD7
RICTOR	RICTOR	MDM2	*NLK*	MAPK14	MAPK1
TSC1	TSC1	IL7R	*TGFBR2*	NLK	CREBBP
RPS6KA3	RPS6KA3	PLK2	*CCNG2*	PIK3R3	MDM2
PRKAA2	PRKAA2	FOXO1	*EP300*	PIK3R1	BCL2L11
ULK1	ULK1	GABARAP	*IL7R*	IRS1	PLK2
PIK3R1	PIK3R1	GRB2	*PLK2*	MAPK3	CCNG2
PIK3R3	PIK3R3	IRS1	*INSR*	BCL2L11	IRS2
ULK2	ULK2	TGFBR1	*FASL*	MAPK1	SMAD2
EIF4B	EIF4B	EGFR	*CCNG2*	CREBBP	KLF2
PRKCB	PRKCB	PRKAA2	*ATM*	PDPK1	GADD45A
PRKCA	PRKCA	NRAS	*SMAD4*	MDM2	AKT2
PTEN	PTEN	SOS1	*S1PR1*	BCL2L11	SGK3
	IGF1	CCNG2	*SMAD2*	IGF1	CCNG2

	CAB39	CCND2	*KLF2*	PLK2	
	MAPK1	ATM	*SMAD3*	TGFBR2	
	RPS6KA2	SETD7		BNIP3	
		STK4		STK4	
		BCL2L11		MAP2K2	
		AGAP2		SIRT1	
		SGK1		CAT	
		PTEN		GABARAP	
		SOD2		TGFB1	
		HOMER1		G6PC3	
		KLF2		CCND1	
		MAPK8		MAPK8	
				PRKAG1	
				AGAP2	
				PRKAA1	
				FOXO3	
				CDKN1A	
				IL10	
				GRB2	
				SGK1	

Table S4: miRNA-Targeted Genes in involved in pathways related to immune function

Bacterial Invasion of Epithelial Cells	
**hsa-miR-27a-3p**	**hsa-miR-92b-3p**
**MET**	CRKL
**ITGB1**	CRK
**CBL**	ITGA5
**DNM2**	CLTC
**CRK**	VCL
**SHC1**	CD2AP
**PIK3CB**	CTNNB1
**CAV1**	CLTA
**SEPT11**	SRC
**PXN**	ARPC5
**CLTC**	
**VCL**	
**RHOA**	
**CD2AP**	
**PTK2**	
**CBLB**	
**PIK3R3**	
**CTNNB1**	
**CTNNA1**	
**DOCK1**	
**PIK3R1**	
**MAD2L2**	
**ARPC1A**	
**RAC1**	
**CDC42**	
**FN1**	
**SEPT2**	
**ARPC4**	
**ARHGEF26**	
**SEPT9**	
**ILK**	

Table S5:

Sequence alignment analysis for the miRBase output sequences: The sequences were aligned (mirbase.org and BLASTn) against those of homologous human and mouse miRNAs.

Query1 sequence *Myotis lucifugus*: UACCCUGUAGAACCGAAUUUGU - 22
| | | | | | | | | | | | | | | | | | | | | | |
Subject sequence *Homo sapiens*: UACCCUGUAGAACCGAAUUUGUG 23 hsa-miR-10b-5p
| | | | | | | | | | | | | | | | | | | | | | |
Subject sequence *Mus musculus*: UACCCUGUAGAACCGAAUUUGUG 23 mmu-miR-10b-5p

Query2 sequence *Myotis lucifugus*: AGGGCUUAGCUGCUUGUGAGCA 22
| | | | | | | | | | | | | | | | | | | | | | |
Subject sequence *Homo sapiens*: AGGGCUUAGCUGCUUGUGAGCA 22 hsa-miR-27a-5p
| | | | | | | | | | | | | | | | | | | | | | |
Subject sequence *Mus musculus*: AGGGCUUAGCUGCUUGUGAGCA 22 mmu-miR-27a-5p

Query3 sequence *Myotis lucifugus*: AGGGACGGGACGUGGUGCAGUGUU 24
| | | | | | | | | | | | | | | | | | | || | | |
Subject sequence Mus musculus: AGGGACGGGACGUGGUGCAGUGUU 24 hsa-miR-92b-5p
| | | | | | | | | | | | | | | | | | || | | |
Subject sequence *Homo sapiens*: AGGGACGGGACGCGGUGCAGU - - -21 mmu-miR-92b-5p

Query4 sequence *Myotis lucifugus*: - GGGGGGCAGGAGGGGCUCAGGG 22
| | | | | | | | | | | | | | | | | | | | | | |
Subject sequence *Homo sapiens*: GGGGGGCAGGAGGGGCUCAGGG 23 hsa-miR-328-5p
| | | | | | | | | | | | | | | | | | | | | | |
Subject sequence *Mus musculus*: -GGGGGCAGGAGGGGCUCAGGG 22 mmu-miR-328-5p

```
Query4 sequence Myotis lucifugus:    AAGUUGCCCGCGUGUUUUUCG   21
                                      | | | | | | | | | | | | | | | | | | | | |
Subject sequence Mus musculus:       AAGUUGCCCGCGUGUUUUUCG   21  mmu-miR-543-5p
                                     |  | | | | | | | | . | | | |       | | | | |
Subject sequence Homo sapiens:      GAAGUUGCC --CAUGUUAUUUUCG 22  mmu-miR-543-5p
Query5 sequence Myotis lucifugus:    CGGGGCCGUAGCACUGUCUGA--   21
                                      | | | | | | | | | | | | | | | | | | | | |
Subject sequence Mus musculus:       CGGGGCCGUAGCACUGUCUGA   21  mmu-miR-128-1-5p
                                      | | | | | | | | | | | | | | | | | | | | |
Subject sequence Homo sapiens:       CGGGGCCGUAGCACUGUCUGAGA 23  hsa-miR-128-1-5p
```
